# Functional outcome after pediatric cerebral cavernous malformation surgery

**DOI:** 10.1038/s41598-023-29472-5

**Published:** 2023-02-09

**Authors:** Laurèl Rauschenbach, Alejandro N. Santos, Thiemo F. Dinger, Marvin Darkwah Oppong, Yan Li, Stephan Tippelt, Christian Dohna-Schwake, Börge Schmidt, Ramazan Jabbarli, Karsten H. Wrede, Ulrich Sure, Philipp Dammann

**Affiliations:** 1 Department of Neurosurgery and Spine Surgery, University Hospital Essen, Essen, Germany; 2 Center for Translational Neuro- and Behavioral Sciences, C-TNBS, University Duisburg Essen, Essen, Germany; 3 Institute of Diagnostic and Interventional Radiology and Neuroradiology, University Hospital Essen, Essen, Germany; 4 Department of Neonatology, Pediatric Intensive Care, Pediatric Neurology, University Hospital Essen, Essen, Germany; 5 Institute for Medical Informatics, Biometry and Epidemiology, University Hospital Essen, Essen, Germany

**Keywords:** Neuroscience, Medical research, Neurological disorders

## Abstract

The purpose of this study was to investigate the functional outcome following surgical resection of cerebral cavernous malformations (CCM) in pediatric patients. We screened our institutional database of CCM patients treated between 2003 and 2021. Inclusion regarded individuals younger or equal than 18 years of age with complete clinical baseline characteristics, magnetic resonance imaging dataset, and postoperative follow-up time of at least three months. Functional outcome was quantified using the modified Rankin Scale (mRS) score and assessed at admission, discharge, and last follow-up examination. The primary endpoint was the postoperative functional outcome. As a secondary endpoint, predictors of postoperative functional deterioration were assessed. A total of 49 pediatric patients with a mean age of 11.3 ± 5.7 years were included for subsequent analyses. Twenty individuals (40.8%) were female. Complete resection of the lesion was achieved in 44 patients (89.8%), and two patients with incomplete resection were referred for successive remnant removal. The mean follow-up time after surgery was 44 months (IQR: 13 – 131). The mean mRS score was 1.6 on admission, 1.7 at discharge, and 0.9 at the latest follow-up. Logistic regression analysis adjusted to age and sex identified brainstem localization (aOR = 53.45 [95%CI = 2.26 − 1261.81], *p* = .014) as a predictor of postoperative deterioration. This study indicates that CCM removal in children can be regarded as safe and favorable for the majority of patients, depending on lesion localization. Brainstem localization implies a high risk of postoperative morbidity and indication for surgery should be balanced carefully. Minor evidence indicates that second-look surgery for CCM remnants might be safe and favorable.

## Introduction

Cerebral cavernous malformations (CCM) are rare neurovascular low-flow lesions that can affect patients of any age, but frequently individuals younger than 18^1–5^. Comparable to adult CCM cases, lesions in pediatric patients can grow and bleed spontaneously, resulting in headaches, focal neurological deficits, or epileptic seizures^2,6–11^. Although the overall incidence of intracerebral hemorrhage (ICH) in young patients is rare, bleeding events can be disabling and severely impact physical and mental health, given the long-life expectancy of children and adolescents^12–14^.

The standard of care for young patients with CCM mainly relies on watchful waiting or neurosurgical lesion removal^15^. Since prospective multicenter data on pediatric CCM patients is missing, clinical management is rarely evidence-based but often personalized and heterogenous, depending on the expertise of the treating physician and distinct characteristics of the affected patient, e.g., patient age, CCM localization, CCM multiplicity, frequency of bleeding events, or clinical condition.

Notably, while watchful waiting implies the risk of successive bleeding with subsequent functional impairment^7,16,17^, operative procedures can be accompanied by significant perioperative morbidity^12,18–20^. Since the available literature is sparse and often controversial regarding the most appropriate treatment option^21,22^, patients, parents, and physicians often face the question of whether to opt for conservative or surgical treatment.

Pediatric trials investigating the efficacy of surgery and risk factors leading to impaired functional outcomes are rare and often limited to small patient cohorts, heterogeneous reporting, and short post-operative follow-up periods^5,7,15^. Moreover, reports on the functional outcome after the subsequent removal of CCM remnants are not existing. Consequently, children and adolescent patients are usually treated according to guidelines for adults, although the appropriateness of this extrapolation is not known. A more profound knowledge of surgical efficacy and risk factors influencing the postoperative outcome could significantly impact current treatment strategies. Therefore, this study aimed to analyze the functional outcome following first or recurrent surgical CCM removal in a pediatric population.

## Methods

### Study design

We conducted this cross-sectional observational single-center study in accordance with the principles expressed in the Declaration of Helsinki and all experimental protocols were approved by an institutional review board (Ethics Committee, University of Duisburg-Essen, Germany, identification board number: 14-5751-BO, 19-8662-BO). Informed consent was obtained from all subjects and/or their legal guardians. Patients were eligible for study inclusion if they were surgically treated in our tertiary care center between 2003 and 2021 and fulfilled the below-mentioned criteria.

### Inclusion criteria

Study inclusion required the following criteria: patients younger than or equal to 18 years of age, diagnosis of CCM, complete baseline characteristics, complete preoperative and postoperative magnetic resonance imaging (MRI) dataset, neuropathological confirmation of CCM, and postoperative follow-up of at least three months.

### Clinical management

The decision for or against surgery was made individually, based on the patient's age, number of previous diseases, neurological burden, number of previous bleeding events, location of lesions, and available scientific evidence^15,21^. Consistent with published data, in the case of multiple CCM only the lesion causative of the clinical symptoms was removed, whereas other innocent lesions remained in situ^23,24^. The decision on surgery was obtained at an interdisciplinary neurosurgical conference. The final decision on whether to operate or not was made jointly with patients and/or parents within a shared-decision making concept, taking into account the individual values and preferences of each patient.

### Data collection

Diagnosis of CCM or CCM-remnants was based on multiplanar MRI, including T1, T2, contrast-enhanced, and susceptibility-weighted or T2*-weighted gradient-echo imaging. An independent neuro-radiologist assessed MRI findings.

Medical records and imaging data were used to assess clinical features, i.e., age, sex, mode of clinical presentation, CCM localization, CCM multiplicity, presence of CCM-associated developmental venous anomaly (DVA), the occurrence of CCM-related hemorrhage, and physical condition. Lesions were defined as deep-seated if they were located subcortically and below the deepest adjacent sulcus. All other lesions were defined as superficial. Axial and sagittal plane T2-weighted images were used to calculate the largest craniocaudal (d_cc_), anteroposterior (d_ap_), and lateral (d_l_) diameter (in mm) of each lesion or the respective hemorrhage. Diameter-based volume (V) of each CCM was assessed according to the ellipsoid formula: V = d_cc_ x d_ap_ x d_l_ x π/6. Neuroimaging underwent automatic segmentation using BRAINLAB® cranial planning software to identify lesions in eloquent brain areas. Eloquent regions were defined as areas associated with language, vision, and sensorimotor processing and included the brainstem, basal ganglia, thalamus, corpus callosum, hypothalamus, insula, dominant hemisphere frontal operculum, dominant hemisphere posterior temporal lobe motor cortex, sensory cortex, or the visual cortex.

In the case of multiple cavernomas on MRI, a full MRI scan of the neuroaxis was usually performed to assess the full extent of the cavernoma burden. Familial cavernomatosis was assumed in cases of cavernoma multiplicity, absence of associated developmental venous anomaly, and/or genetically confirmed CCM1, CCM2, or CCM3 mutation, and/or known affected relatives^25–27^.

The degree of neurological disability was obtained using the modified Rankin Scale (mRS) score. The outcome was considered favorable, if patients had a mRS score less than or equal to 2 and unfavorable if the mRS score was greater than 2. We assessed mRS scores at the time of diagnosis, preoperatively, and postoperatively at the last follow-up. A minimum one-point increase on the mRS score compared to the preoperative score was defined as neurological deterioration. The presentation mode was obtained in all patients according to established reporting standards^6^: CCM-related epilepsy (CRE), symptomatic ICH, non-hemorrhagic focal neurological deficit, non-hemorrhagic CRE, or asymptomatic. Symptomatic ICH was classified according to reporting standards as follows: acute or subacute onset of neurological symptoms related to the anatomical region of the lesion accompanied by radiological evidence of acute bleeding of the CCM on a recent MRI^6^. Postoperative seizure control was classified according to the commission report of the International League Against Epilepsy (ILAE) on postoperative outcomes after epilepsy surgery^28^. Definitive seizure control was classified as ILAE class 1, while improvement was classified as ILAE classes 2, 3, and 4. Diagnosis of CRE was obtained through an experienced epileptologist and required an electroencephalography-based association of epilepsy and circumscribed cavernoma. The extent of resection was diagnosed on MRI at the first postoperative follow-up examination and defined as complete or incomplete removal.

The primary endpoint of this study was to assess the postoperative neurological outcome. The secondary endpoint was to investigate risk factors influencing the outcome.

### Statistical analyses

Statistical testing was performed using SPSS 27 (IBM Corp.) and results were visualized using PRISM 9.0 (GraphPad Software). Results were considered statistically significant at an alpha-level of less than 0.05. Data were tested for normal distribution by performing a Shapiro–Wilk test. First, the association between postoperative neurological deterioration and candidate prognostic factors was assessed using univariate analysis. Univariate analyses were performed to determine predictors of postoperative deterioration. The Chi-Square test (sample size more than 5) or the Fisher exact test (sample size less than or equal to 5) were used for dichotomized variables. Continuous variables were tested with the Student’s t-Test (normally distributed data) or Mann–Whitney-U test (non-normally distributed data). Logistic regression analyses, adjusted for age and sex, were performed to confirm predictors of postoperative deterioration.

## Results

### Patient demographics and outcomes

A total of 49 pediatric patients were considered for analysis. The mean age was 11.3 ± 5.7 years, and 20 individuals (40.8%) were female. Intracerebral distribution of CCM lesions was rather heterogeneous. The majority of patients (28.6%) revealed temporal lobe CCM, followed by frontal lobe (22.4%), brainstem (16.3%), occipital lobe (14.3%), parietal lobe (12.2%) and cerebellar (6.1%) localization. Forty patients (81.6%) suffered from ICH at diagnosis. CCM-associated DVA was observed in 8 patients (18.2%), and CCM-related epilepsy was present in 25 (51.0%) individuals. Four patients (8.7%) were asymptomatic at the time of diagnosis. In the majority of cases, CCM appeared as solitary lesions, but 17 patients (36.2%) presented with multiple CCM lesions. Of those, 8 patients (47.1%) revealed ≥ 5 intracerebral lesions.

**Figure 1:**
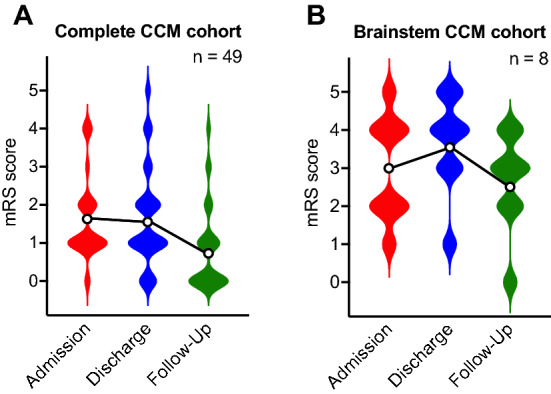
Illustration of longitudinal mRS scores preoperatively at admission, postoperatively at discharge and postoperatively at last follow-up examination. Data is presented as violin plots. Circles indicate mean values. (**A**) Data of the whole CCM cohort. (**B**) Data of the brainstem CCM cohort. *CCM* cerebral cavernous malformation, *mRS*: modified Rankin Scale.

The median follow-up time after surgery was 44 months (IQR: 13 – 131). Complete resection of the lesion was achieved in 44 patients (89.8%). Five patients revealed incomplete resection, and two individuals were referred for subsequent remnant removal. At admission, 41 patients (83.7%) were in good clinical condition, revealing a mRS score of less or equal than 2. With a mRS score of less or equal than 2 after complete resection, a favorable outcome was observed in 44 patients (89.8%), and most individuals (91.8%) revealed improved or unchanged scores at last follow-up. The mean mRS score was 1.6 on admission, 1.7 at discharge, and 0.9 at latest follow-up, respectively. Detailed cohort characteristics are summarized in Table 1. Longitudinal distribution of mRS scores is presented in Fig. 1A. Postoperative analysis of epilepsy patients showed that 18 patients (72.0%) were seizure-free (ILAE class 1) and another 2 patients (8.0%) benefited from surgery in terms of seizure frequency (ILAE class 2–4). Univariate analysis revealed no association between postoperative seizure freedom and age, sex, lesion side, temporal or extratemporal lesion localization, lesion volume, lesion depth, eloquent localization, CCM multiplicity, or existence of DVA (*p* > .05).

**Table 1:** Univariate analysis of demographic, clinical and anatomical predictors for postoperative deterioration.

Parameter	No postoperative deterioration (n = 45, 91.8%)	Postoperative deterioration (n = 4, 8.2%)	*p*	OR	95%CI
Age (years), mean ± SD	11.1 ± 5.9	13.3 ± 2.2	.480^a^	N/A	N/A
Female sex, n (%)	19 (42.2%)	1 (25.0%)	.636^b^	.46	.04–4.73
Lesion side, n (%)			.999^b^	N/A	N/A
• Left	22 (48.9%)	2 (50.0%)			
• Right	20 (44.4%)	2 (50.0%)			
• Midline	3 (6.7%)	0 (0.0%)			
Lesion localization, n (%)			**.026**^b^	N/A	N/A
• Supratentorial	37 (82.2%)	1 (25.0%)			
• Cerebellar	3 (6.7%)	0 (0.0%)			
• Brainstem	5 (11.1%)	3 (75.0%)			
Lesion volume in cm^3^, mean ± SD	4.39 ± 4.53	5.76 ± 4.39	.564^a^	N/A	N/A
Deep-seated lesion, n (%)	28 (62.2%)	4 (100.0%)	.284^b^	1.14	1.00–1.30
Eloquent localization, n (%)	18 (40.0%)	3 (75.0%)	.301^b^	1.13	0.93–1.36
Multiple CCM (≥ 2 CCM), n (%)	16 (35.6%)	1 (25.0%)	.999^b^	.60	.06–6.30
DVA^§^, n (%)	7 (17.5%)	1 (25.0%)	.566^b^	1.57	.14–17.42
CRE, n (%)	24 (53.3%)	1 (25.0%)	.349^b^	.29	.03–3.02
Recurrent bleeding in history, n (%)	12 (26.7%)	2 (50.0%)	.568^b^	2.75	.35–21.75
ICH at presentation, n (%)	37 (82.2%)	3 (75.0%)	.569^b^	.65	.06–7.07
Asymptomatic at presentation*, n (%)	4 (9.5%)	0 (0.0%)	.999^b^	.91	.82–1.00
Complete resection, n (%)	41 (91.8%)	3 (75.0%)	.359^b^	.25	.02–3.28
mRS preoperative, n (%)			**.039**^b^	N/A	N/A
• 0	2 (4.4%)	1 (25.0%)			
• 1	26 (57.8%)	0 (0.0%)			
• 2	9 (20.0%)	3 (75.0%)			
• 3	2 (4.4%)	0 (0.0%)			
• 4	6 (13.3%)	0 (0.0%)			
• 5	0 (0.0%)	0 (0.0%)			
• 6	0 (0.0%)	0 (0.0%)			
mRS postoperative, n (%)			**< .001**^b^	N/A	N/A
• 0	25 (55.6%)	0 (0.0%)			
• 1	13 (28.9%)	1 (25.0%)			
• 2	5 (11.1%)	0 (0.0%)			
• 3	2 (4.4%)	1 (25.0%)			
• 4	0 (0.0%)	2 (4.4%)			
• 5	0 (0.0%)	0 (0.0%)			
• 6	0 (0.0%)	0 (0.0%)			

A minimum one-point increase on the mRS score compared to the preoperative score was defined as deterioration. Changes in mRS refer to comparison between mRS at admission and final follow-up.

*P* values marked with bold indicate statistically significant differences between the groups.

^a^Student’s t-test or Mann–Whitney-U test.

^b^Chi-Square test or Fisher exact test.

*CCM* cerebral cavernous malformation, *CRE* CCM-related epilepsy, *DVA* developmental venous anomaly, *ICH* CCM-related intracerebral hemorrhage, *mRS* modified Rankin Scale, *N/A* not applicable, §: 5 patients missing, *: 3 patients missing.

### Predictors of postoperative outcome

We found no significant correlation between extent of resection and lesion localization (brainstem, *p* = .18, frontal lobe, *p* = .57, parietal lobe, *p* = .99, temporal lobe, *p* = .99, occipital lobe, *p* = .55, cerebellar, *p* = .28). Univariate analysis indicated association between surgery for brainstem CCM and worse mRS scores after surgery (OR = 24.03 [95%CI = 2.08–277.21], *p* = .011). Logistic regression analysis adjusted for age and sex identified brainstem localization (aOR = 53.45 [95%CI = 2.26–1261.81], *p* = .014) as statistically significant predictor for postoperative deterioration. Data is presented in Table 2. Longitudinal distribution of mRS scores after brainstem CCM removal is presented in Fig. 1B.

**Table 2:** Logistic regression analysis adjusted for age and sex assessing baseline characteristics and risk of postoperative deterioration.

Parameter	*p*	aOR	95%CI
Brainstem localization	**.014**	53.45	2.26–1261.81

A minimum one-point increase on the mRS score compared to the preoperative score was defined as deterioration. Changes in mRS refer to comparison between mRS at admission and final follow-up. *P* values marked with bold indicate statistically significant differences between the groups.

### Safety of successive remnant removal

As stated above, five patients (10.2%) revealed lesion remnant. Out of these, 2 (40%) underwent subsequent remnant removal. After second-look surgery, one patient temporarily deteriorated but recovered during follow-up, while the second patient remained with an unchanged mRS score over time. Regarding the remaining three patients, one improved and two remained unchanged. None of the patients with incomplete resection status experienced symptomatic recurrent ICH during follow-up. More details are illustrated in Table 3.

**Table 3:** Illustration of longitudinal mRS scores at diagnosis, after first and second surgery, and at last follow-up.

Patient #	Preoperative mRS score at diagnosis	Postoperative mRS score after first surgery	Postoperative mRS score after second surgery	Postoperative mRS score at last follow-up
Patient #1	2	3	4	3
Patient #2	1	1	1	0
Patient #3	1	2	N/A	1
Patient #4	2	2	N/A	2
Patient #5	4	4	N/A	2

*mRS* modified Rankin Scale, *N/A* not applicable.

## Discussion

Diagnosis of CCM often affects individuals younger than 18 years of age. In this population, these lesions represent a considerable source of spontaneous ICH^1–4^. The value of neurosurgical lesion removal is still discussed controversially, balancing the lifetime risk of spontaneous bleeding events and the risk of surgery-related morbidity^21,22^. Moreover, CCM research in children and adolescents is often limited to small sample size data due to the rarity of the disease. The functional outcome after lesion removal and the implications of second-look surgeries for remnant removal are largely unknown in this context. To this extent, a deeper knowledge of the effect of surgery and re-surgery, and risk factors influencing postoperative outcomes could significantly impact current management. Therefore, we aimed to analyze the functional outcome following the first and the second surgical removal of CCM in a pediatric population and to assess postoperative deterioration predictors.

### Predictors of postoperative outcome

Our analysis, adjusted to age and sex, identified brainstem localization as a predictor for postoperative deterioration, while non-brainstem localization was not associated with worse outcomes. In accordance with large studies on adult patients and some small-sample pediatric trials, surgery for supratentorial and cerebellar lesions is favorable and safe^2,3,9,14,18,19,29–31^, while removal of brainstem CCM remains complex and is associated with a significant risk of postoperative morbidity^12,20,21,32^. In our study, the mean mRS value at admission was 2.0, while in the largest available pediatric brainstem CCM trial of Li and colleagues the authors describe a mean mRS value of 3.0^20^. This might indicate different clinical management strategies for pediatric patients with brainstem CCM and highlight the poor consensus regarding indications for surgery. Nevertheless, in both studies, follow-up mRS values were lower than at admission, underlining the overall benefit of brainstem CCM removal for the majority of patients. Our findings corroborate the risky nature of brainstem CCM removal and suggest a careful balance between surgery and watch-and-wait strategies. Nevertheless, our work has studied surgically treated patients and no conclusion can be drawn about the advantages or disadvantages of surgical versus conservative treatment. Moreover, our cohort comprises a small number of brainstem cases and represents a severe simplification, since brainstem CCM is a heterogeneous group of lesions with different sizes, locations, and clinical courses.

### Extent of resection and surgery for CCM remnants

According to the existing literature, total resection of the lesion generally ensures good clinical outcomes, mainly depending on lesion localization^9,12,16^. In their previously published study, Li and colleagues focused on the surgical outcome of pediatric patients with brainstem CCM^20^. The authors obtained fair outcomes after lesionectomy but only a small number of fully recovered patients. Moreover, they found a better outcome when complete removal was achieved, reducing the risk of postoperative rebleeding of remnant CCM tissue. These findings were similar in the trial of Gross and colleagues, assessing the surgical outcome of pediatric patients with supratentorial CCM^9^. According to the authors, CCM remnant can lead to postoperative rebleeding, while complete resection is a predictor of good postoperative recovery and eliminated risk of recurrent bleeding events. Although being too small for statistical testing, our found no differences in neurological outcome after obtaining complete or incomplete CCM resection. Although incompletely resected CCM patients did not reveal postoperative bleeding in the follow-up period, our findings are in accordance with the conclusions of Li et al. and Gross et al. and indicate that second-look surgeries might be favorable to avoid rebleeding events.

### External validity

Compared to other studies investigating CCM in pediatric patients, our cohort seems to be representative in terms of patient characteristics^9,12,20^. Compared to the study of Gross and colleagues, which is the largest available trial for supratentorial lesions, the baseline data of both patient cohorts were similar, with 59.2% versus 57% being males, mean age of 11.3 versus 11.8 years, 8.7% versus 5% asymptomatic patients at diagnosis, and 89.8% vs 98% patients with complete resection results^9^. This observation increases the external validity of our reported results. Nevertheless, higher mRS values at admission in the study of Li and colleagues indicate, that CCM populations can differ due to missing consensus in clinical management^20^. This might narrow the overall comparability of study cohorts. Compared to the recently published meta-analysis of Gao and colleagues, which is the largest available study on surgery in CRE children, our cohort reveals similar outcomes, with 72.0% versus 66.7–100.0% seizure freedom^33^.

### Strengths, limitations and perspectives

Although spontaneous ICH in pediatric patients is mainly related to CCM disease, the majority of symptomatic lesions affect adult individuals^34^. Such numbers make the realization of single-center trials with large cohorts difficult. Several groups have contributed valuable data in the past, and our study is methodologically limited and not superior to other studies due to the small number of cases, the heterogeneity of data, and the retrospective design. Moreover, we present data obtained at a tertiary referral center and no population-based data. This could lead to information and selection biases. In particular, the selection of patients for surgery is subject to substantial bias, as many different features influence clinical decision-making (e.g., eloquent or deep-seated lesion localization) and the allocation of patients to surgery or conservative treatment. Despite these limitations, our report presents novel data on a rare disease and, together with other studies, might contribute to improved patient care. Since pediatric cavernoma disease is heterogeneous with varying degrees of prognosis, there is an ongoing need for larger case series and meta-analyses examining the outcomes after surgery in different brain regions. Such a subgroup analysis was not feasible in our study, due to the small number of cases.

## Conclusion

Our study confirms the safety and feasibility of surgical CCM removal in pediatric patients. Lesion location should mitigate decisions towards or against surgery. Little evidence does indicate that second-look surgery for CCM remnants might be safe and valuable, but more detailed studies are needed to confirm this assumption. 

## Data Availability

The datasets generated during and analyzed during the current study are available from the corresponding author upon reasonable request.

## Abbreviations

CCM: Cerebral cavernous malformation
DVA: Developmental venous anomaly
ICH: Intracerebral hemorrhage
MRI: Magnetic resonance imaging
mRS: Modifies Ranking Scale
